# Client experiences with antenatal care waiting times in southern Mozambique

**DOI:** 10.1186/s12913-019-4369-6

**Published:** 2019-08-01

**Authors:** Estelle Gong, Janeth Dula, Carla Alberto, Amanda de Albuquerque, Maria Steenland, Quinhas Fernandes, Rosa Marlene Cuco, Sandra Sequeira, Sérgio Chicumbe, Eduardo Samo Gudo, Margaret McConnell

**Affiliations:** 1 Department of Global Health and Population, Harvard T. H. Chan School of Public Health, Boston, MA USA; 2 Instituto Nacional de Saúde, Ministério da Saúde, Maputo, Mozambique; 3 Pontifical Catholic University of Rio de Janeiro, Rio de Janeiro, Brazil; 4 Population Studies and Training Center, Brown University, Providence, RI USA; 5 National Directorate of Public Health, Ministry of Health, Maputo, Mozambique; 6 Department of International Development, London School of Economics, London, UK

**Keywords:** Quality of care, Antenatal care, Maternal and child health, Health services

## Abstract

**Background:**

Antenatal care (ANC) provides a range of critical health services during pregnancy that can improve maternal and neonatal health outcomes. In Mozambique, only half of women receive four or more ANC visits, which are provided for free at public health centers by maternal and child health (MCH) nurses. Waiting time has been shown to contribute to negative client experiences, which may be a driver of low maternity care utilization. A recent pilot study of a program to schedule ANC visits demonstrated that scheduling care reduces waiting time and results in higher rates of complete ANC. This study aims to explore client experiences with waiting time for ANC in standard practice and care and after the introduction of appointment scheduling.

**Methods:**

This study uses a series of qualitative interviews to unpack client experiences with ANC waiting time with and without scheduled care, in order to better understand the impact of waiting time on client experiences. Thirty-eight interviews were collected in May to June 2017 at three pilot study clinics in southern Mozambique, with a focus on two paired intervention and comparison facilities sharing similar facility characteristics. Data were analyzed using inductive thematic analysis methods using NVivo software.

**Results:**

Clients described strong motivations to seek ANC, pointing to the need to address convenience of care, and highlighted direct and indirect costs of seeking care that were exacerbated by long waiting times. Direct costs include time and transport costs of going to the clinic, while indirect costs include being unable to fulfill household and work obligations. Other barriers to complete ANC utilization of four or more visits include transport costs, negative provider experiences, and delayed ANC initiation, which limit the potential number of clinic contacts.

**Conclusions:**

Findings demonstrate that the scheduling intervention improves the client experience of seeking care by allowing women to both seek ANC and fulfill other productive obligations. Innovation in healthcare delivery should consider adapting models that minimize waiting times.

**Electronic supplementary material:**

The online version of this article (10.1186/s12913-019-4369-6) contains supplementary material, which is available to authorized users.

## Background

A crucial opportunity to improve maternal and child health outcomes is antenatal care (ANC), which includes a range of critical services during pregnancy for health education, disease monitoring, prevention, and treatment [1, 2]. In Mozambique, ANC services are available at the primary health care level, with over 1000 primary care facilities across the country [3]. Maternal and child health (MCH) nurses primarily deliver these services, which are free of cost to clients [4, 5].

Despite ANC’s wide availability in Mozambique, infant and maternal mortality rates remain high at 53 infant deaths per 1000 live births [6] and 489 maternal deaths per 100,000 live births [7]. It is possible that the full benefits of ANC may not be realized, in part due to inadequate quality of care and low service utilization [8, 9]. Recent efforts by the Ministry of Health (MOH) have begun to address the technical quality of ANC, particularly through the implementation and evaluation of a supply kit and training intervention aimed at increasing key service delivery [8, 10, 11]. However, while improving technical quality of care is critical, an under-explored dimension of quality is the client experience and its relationship with the uptake of care [12].

The opportunity for the client experience to impact care utilization is implicit in research identifying barriers and facilitators to accessing routine care. For example, interviews with HIV and ANC clients at public clinics in Mozambique, Ethiopia, Tanzania, and South Africa identified negative client experiences as barriers to care. These experiences included clinic overcrowding, negative interactions with providers, and long wait times [8, 13–17]. Based on these findings, addressing negative experiences could increase access to and uptake of care. However, until recently, there was little evidence demonstrating and measuring the relationship between client experience and care utilization. The *Lancet Global Health* Commission on High-Quality Health Systems, launched in 2017, calls specifically for more research on patient experience as a possible driver of healthcare use [18]. With the 2016 update to the World Health Organization ANC guidelines that increases the number of recommended ANC visits from four to eight, the need for increased ANC utilization is especially relevant [2].

This study accompanies an investigation by Steenland et al. [19] that evaluated the effect of a pilot ANC scheduling intervention on waiting time and ANC utilization using quantitative data collected as part of this study. Prior to the intervention, women arrived early in the morning, often before the clinic opened, and queued for hours before they were seen [8, 19]. After six months of the intervention, average ANC waiting time had halved from three hours, and the percentage of women receiving four or more visits was estimated to increase by sixteen percentage points [19]. Results from this pilot study provide promising early evidence that scheduling care may reduce waiting time and increase utilization of ANC. The present study aims to explore clients’ motivations and preparations for attending ANC, how waiting time factors into deciding and planning to attend ANC, and provide a sense of clients’ experiences and perceptions of the scheduling system.

### Study context

From November 2016 to June 2017, an appointment scheduling intervention was implemented at four health centers in southern Mozambique: two clinics in urban areas of Maputo province, and one clinic each in rural areas of Inhambane and Gaza provinces. A fifth clinic, also in urban Maputo, was monitored as a comparison. At all study clinics, ANC was provided among other maternal and child health services, all at no cost to clients [4].

For women seeking care in these areas, and whose occupations include selling goods, domestic work, and agriculture [20], distance and cost-related challenges remain barriers to care [21]. In 2015, approximately half of women received the MOH-recommended four or more ANC visits (55%) [4, 22] and 70% chose to deliver at a health facility [22]. Provincial estimates were higher for ANC coverage of four or more visits, with 81% in Gaza Province, followed by 74% in Maputo Province and 62% in Inhambane Province [22].

At health centers prior to the intervention, and at the comparison clinic, ANC was organized as follows: most clients would arrive early in the morning before the clinic opened and stack their ANC booklets in the order of arrival. When the clinic opened, MCH nurses would begin calling clients for consultations based on booklet order. Organization of first time ANC visits varied from clinic to clinic, with some clinics giving priority to first time visits while others did not.

At intervention clinics, ANC was reorganized to reserve the first hours of the day (from 7:30 A.M. until 10 A.M.) for first time ANC visits and the remainder of the day (from 10 A.M. until 3:30 P.M.) was scheduled for return ANC visits. MCH nurses were provided an appointment scheduling book, where each page allowed nurses to schedule approximately five clients per hour between 10 A.M. and 3 P. M on a specific date. Clients were instructed to return for their next ANC visit at the specified date and time, and appointment details were recorded on a scheduling card stapled to the client’s ANC booklet that was brought to every appointment.

## Methods

Semi-structured interviews were conducted with clients at three study clinics, including two clinics in Maputo Province chosen because of similar facility characteristics, one of which was assigned to the intervention (Boane) and one was not (Machava II). The third clinic (Chissano), where the scheduling intervention was also implemented, was chosen because it represented a rural service area. This site selection aimed to capture client experiences with waiting time and allow for reflection on the new system of scheduling appointments. In total, 38 semi-structured in-depth interviews lasting 30 to 60 min were collected from May to July 2017, at the conclusion of the six-month pilot. While data collection focused on Boane (*n* = 17) and Machava II (*n* = 19), the two paired intervention and comparison clinics, 2 interviews were conducted at Chissano, the rural clinic in Gaza Province. These interviews were included in analysis to capture a greater range of experiences and to enrich data. Though not available for interview participants, client demographic characteristics for each facility were collected at baseline for the quantitative evaluation and can be found in, Additional File 1: Table S1 [19].

To obtain a wide range of client experiences in a limited data collection period, participants were recruited with purposeful random sampling [23, 24]: data collection occurred at intervention and comparison sites on different days of the week to capture variation in weekday volume trends, and clients were recruited at random at the conclusion of their ANC appointment without prior knowledge of their experience. Inclusion criteria were that women were at the clinic for their ANC appointment, were eight or nine months pregnant, and were 18 years or older. The sample was restricted to this gestational age to allow women to reflect on their experience seeking ANC during their most recent pregnancy, and to ensure the sample represented a wide range of ANC seeking experiences, as the majority would have initiated some ANC by this stage [21].

### Data collection

Interviews were collected using a semi-structured Portuguese interview guide that was developed for this study (Additional File 2). Questions were open ended and invited participants to discuss how they decided and prepared to attend ANC that day and their experiences at the clinic. For participants at the intervention clinics, they were also asked about their experience with the scheduling system. No pre-existing framework informed the interview guide, and participants were instead probed to elaborate on statements of interest.

Interviews took place in a private and seated outdoor area that was on clinic grounds but separated from main clinic activities. Before participants provided formal consent, researchers explained the purpose of the interview, that responses were confidential, and that participation was voluntary. Pairs of researchers conducted the interview, with one researcher conducting the interview in Portuguese, Changana, or Xitshwa, and the other researcher taking notes.

Interviews were audio recorded with participant permission, transcribed into Portuguese, and translated into English. Interviews conducted in Changana or Xitshwa were transcribed directly into Portuguese. The quality of transcription and translation was checked by comparing against a sample of independently transcribed and translated interview segments.

### Data analysis

A team of three researchers analyzed transcripts using an inductive thematic analysis approach informed by Grounded Theory coding methodology. EG developed an initial codebook for clients using line-by-line coding, developing an action-oriented code for each participant statement (e.g. Choosing facility based on proximity and routine) [25]. Approximately 10% of transcripts were coded line-by-line, which were then categorized into focused codes (e.g. Facility choice).

**Fig. 1:**
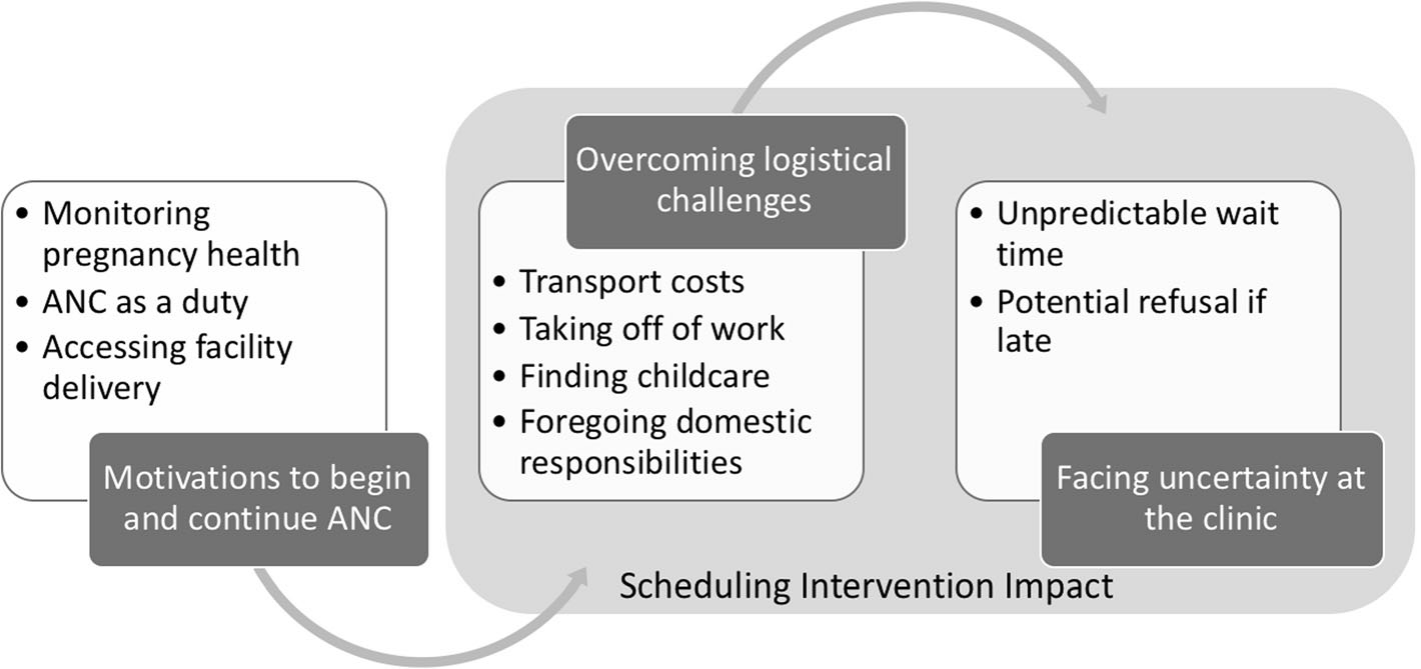
Conceptual map describing the client experience in deciding and planning to attend antenatal care

JD and CA applied the initial codebook of focused codes to another 10% of transcripts, and the team discussed differences and reached a consensus or refined the codebook appropriately. EG applied the codebook to the remaining transcripts and used constant comparison in developing higher order theoretical codes that captured broad themes (Fig. 1). The group agreed on thematic saturation given the interview sample and that additional data collection was unnecessary. Interviews were analyzed using NVivo 11 [26].

### Reflexivity

Research team members collecting and analyzing data held a variety of perspectives. JD and CA, who conducted interviews, were also part of the pilot study research team and were thus familiar with the clinic staff and day-to-day context of the clinics and intervention. Additionally, they are from the city of Maputo and trained in medicine and medical anthropology, respectively. EG, who took notes during the interviews and led data collection and analysis, is an American public health researcher who was less familiar with the study and cultural context. The diverse perspectives of this research team aimed to address both insider and outsider bias in data collection and analysis.

### Study approval

This qualitative study was approved by the Harvard T. H. Chan School of Public Health’s Institutional Review Board (IRB16–0344) and the Mozambican Ministry of Health’s Comité Institucional de Bioética do Instituto Nacional de Saúde.

## Results

Long waiting times for ANC negatively impacted client experiences in seeking care, and the scheduling system improved the client experience by reducing time spent at the clinic. Clients at both intervention and comparison facilities were motivated to seek ANC but were hindered by direct (e.g. time, food, and transport) and indirect (e.g. foregoing other responsibilities) costs of going to the clinic. In their experiences without a scheduling mechanism, clients faced unpredictable and long wait times and uncertainty about whether they would be seen at all. The scheduling system addressed some costs of attending ANC by addressing logistical challenges and reducing uncertainty around when clients received care.

## Client perspectives

Figure 1 describes the client experience in deciding and planning to attend antenatal care appointments. Clients discuss attending ANC as a duty and are motivated to seek care to monitor the baby’s progression and to protect against illness. To get to appointments, clients overcome logistical challenges to travel to and spend hours at the clinic. At the clinic, clients still face uncertainty in how long they will wait. For some, not arriving early enough can result in refusal. The scheduling system mainly impacts clients by allowing them to more easily address logistical issues and reduce uncertainty at the clinic.

### Motivations – deciding to begin and continue ANC

Clients at both intervention and comparison clinics viewed ANC as an opportunity to monitor pregnancy progress and avoid poor health outcomes, and attending ANC was viewed as an obligation. Additionally, opening and maintaining an ANC record was a way to access facility delivery.

#### Controlling the pregnancy

Women came to the clinic for ANC because it was the control date for their pregnancy. Maintaining control over one’s pregnancy included being weighed and measured to track the progress of their pregnancy, protecting themselves and their child from disease through testing and receiving preventative medicines (such as antimalarials and antiretrovirals), and having the opportunity to consult with nurses.

*Because [ANC] is the only way I’ll know if the baby is doing well or not. If the baby is growing well or not. If the heart is beating or not beating. Because sometimes the baby doesn’t move like they should and we don’t know. – A12, comparison clinic*

*It’s good to come to the [ANC consultation] because we have to be monitored. I don’t know everything, and after I realized I was pregnant, I shouldn’t stay at home far from the hospital and from the nurses. You always have to be monitored. And it’s really good and important to seek medical care and see how you are doing. – B18, intervention clinic*

*There are many diseases. Many diseases that a child can catch. Look, I know that there are kids that are born – some are HIV positive. There are children who are born with a cold. There are kids that are born with a lot of illnesses. But when you come to the hospital, they tell you that you have to do this, do that, so your children will not get an illness. I doubt that if you comply with what they tell you that your child will be born ill. — B16, intervention clinic*

#### Importance and frequency

Clients emphasized the importance of seeking ANC, describing it as an obligation or responsibility. The importance of ANC also influenced the frequency at which clients decided to attend ANC. When clients began ANC, which could be delayed until the second or third trimester, they preferred monthly appointments. As delivery neared, some expressed the need for additional visits.

*[Attending ANC is] not a challenge, it’s a responsibility that everyone should have, it’s an obligation, and it’s not much. It’s a responsibility. – A13, comparison clinic*

*A woman should come to the appointment from the sixth month of pregnancy because at that time you know you are going to the appointment and everyone can see that you’re pregnant. Imagine that you start going to the [ANC] with two months pregnant, it’s too early and people will start thinking it’s a one-year pregnancy, it’s too much. At least open your file with six months and come to the [ANC] four or five times. – B8, intervention clinic*

*I think [ANC visits] could be once a month during the first months. But from the 7th or 8th month you could do it every 2 weeks or once a week – because when you’re almost about to give birth the person might need more control. – B3, intervention clinic*

#### Opening and maintaining the record

A crucial function of seeking ANC is the act of opening the record or file, or enrolling, at the clinic to gain access to facility delivery. From personal experiences or hearing stories from others, clients believed that they could be turned away from the facility during labor if they did not have an ANC record. Additionally, if clients were not compliant with their ANC appointments, they faced potential criticism from MCH nurses. Thus, in addition to seeking ANC for its services and to gain knowledge, clients also complied with ANC to avoid nurse criticism and plan for delivery at a health facility.

*I came to open my file because I think that it’s a woman’s right that when she’s pregnant she should go to the hospital, open the file and have the appointment, so that it’s easier to give birth. Because if you don’t do that, not open the file, what will they say on the delivery date? – A16, comparison clinic*

*[Nurses] will complicate things on the day of delivery [if you do not come to ANC]. They could say to you ‘You’re coming to do what here in the hospital? Why didn’t you come to do your consultations?’— B16, intervention clinic*

### Overcoming logistical challenges and the costs of waiting

In the days leading up to their appointment date, clients begin making arrangements for transport and the responsibilities they cannot carry out on the day of the appointment. Planning to attend ANC incurs direct and indirect costs to clients in both intervention and comparison contexts. However, longer wait times at the comparison clinic often exacerbate the impact of these costs.

Direct costs include time, money for transport and potential food costs. Early mornings and hours spent waiting results in skipped meals, and clients either pack food ahead of time or purchase food if they have the money. For many, purchasing food from nearby vendors is not an option when transportation funds are already scarce, and they may wait hours without eating.

*You’re pregnant and you leave the house and sometimes you think you’ll eat later and sometimes you don’t even have a snack. And if you have 10 meticais for the bus or 20 meticais for the bus and you leave home and you arrive and sit and wait and you arrive [early], someone can sit like they are sick. But you aren’t sick, you’re just weak because you’re hungry. – A17, comparison clinic*

Indirect costs include hours, and often the whole day, of responsibilities foregone for each ANC appointment day. Responsibilities include formal or informal employment, childcare, and other household tasks. While some clients rely on neighbors and family to take over childcare and household responsibilities, others are forced to choose between those responsibilities or the ANC appointment. Long waiting times increase these costs and make attending ANC more challenging for clients.

*[Waiting] doesn’t feel that great, like I’ve said before I work. I don’t feel comfortable asking [work] to come to the appointment and take the whole day … I can’t ask to come to the hospital and then give up because I have to go back to work. It’s worth it to burn that day that [nurses] attended me and go back to work the next day. – A11, comparison clinic*

*When I wake up [and] I see that there aren’t chores to do, I come to the hospital. Last Friday I wanted to come but it wasn’t possible. I had to go get water, do the dishes, clean the house. Today I asked my mom to stay home so I could be here. – B2, intervention clinic*

*For me, I don’t like to leave my son alone. When I take too long [at the clinic], he doesn’t go to school. Now that I’m [pregnant] I don’t know if he goes … How much time will I have to stay here while he’s all alone at home? – A10, comparison clinic*

### Facing uncertainty at the clinic

Clients face significant financial and personal costs in accessing ANC, yet they face uncertainties even after they arrive to the clinic. At the comparison clinic, it was difficult for clients to predict how long they would be waiting or if they would be seen at all. The norm was to arrive early in the morning to minimize wait times, and it was also acknowledged that not arriving early enough could result in being turned away.

*In all honesty it’s not possible to predict the time that I’ll leave the hospital, but we always have in mind that if you arrive early, you’ll be seen early and return early. –A7, comparison clinic*

*On Friday, I wanted to [come here], but I slept in, so when I woke up it was already 7:00AM and I couldn't come anymore because they wouldn't have attended me. Therefore, I had to stay home and came today. – A2, comparison clinic*

### Client experiences with the scheduling system intervention

As depicted in Fig. 1 by the box labeled *Scheduling Intervention Impact,* the scheduling system affected mainly logistical challenges and uncertainty at the clinic.

#### Improved organization of time

With appointments scheduled for late morning and early afternoon at intervention clinics, time in the morning was now available for clients to complete necessary tasks before going to the clinic. Clients reported being able to accomplish both attending ANC and other responsibilities such as chores in the morning and sending children to school.

*In that old system you would get up early and come here and wait until 2 P.M. while you didn’t do anything at home. Now you can plan, you wake up at 5 A.M., clean the backyard, do the dishes, clean the house, then make breakfast for the children who then leave, and you stay preparing the curry [dinner]. – B12, intervention clinic*

One client expressed the main accomplishment of the scheduling system as making her appointment days more predictable, regarding both when she leaves for the clinic and when she returns home.

*I think it’s better with the booking system … because when we know what time we’ll be seen we can schedule the time we leave the house and what time we’ll get back – we more or less know our schedule for the day. – B3, intervention clinic*

#### Remaining uncertainty

While clients appreciated the ability to arrive later in the morning, appointments could still be delayed with the pilot scheduling system. For clients who arrived past their appointment time, or not adequately ahead of time, it was possible to be refused.

*It was only last month that I had to be here at 12 P.M. and on the card it was written 12 P.M. to 1 P.M. I only got here at 12 P.M. because I was waiting for my daughter who goes to school in the morning. They already had made the collection of the cards and told me to come back the next day … They said, ‘You can’t be late again, if it says 12 P.M. you have to be here at 11 A.M.’ – B13, intervention clinic*

Though there was some confusion around the scheduling system, clients still complied and thought positively of the new system. They also often attributed any delays to crowded facilities rather than the nurses themselves, who clients believed were doing the best that they could.

*It’s not like before when we didn’t have this [appointment] card. We would come here early and wait. It took a long time to get home. With this card it got better … I know the nurses can’t do everything we want because there’s just too many of us. If they tell me to come at 1 P.M. it doesn’t mean I’ll be seen at the time. But I know it is not her fault. – B1, intervention clinic*

## Discussion

This qualitative investigation, which complements a pilot trial of an ANC scheduling intervention to reduce waiting times, sought to explore client experiences with ANC waiting times in settings with and without appointment scheduling. Our findings illustrate how long waiting times pose direct and indirect costs to clients seeking care, and that a scheduling intervention mitigates costs by improving predictability of accessing ANC. Further, while our findings also identified other ANC barriers that are consistent with past literature, such as transportation costs and negative provider experiences, lack of knowledge on the importance of ANC was not a barrier in this context. Instead, clients held strong beliefs about the need for ANC during pregnancy, suggesting that other factors such as convenience of care may be important drivers of utilization to consider when designing interventions.

Client experiences with waiting times demonstrated the time costs of seeking care, both in terms of direct costs of spending uncomfortable hours at the clinic and indirect costs of foregoing responsibilities such as child care, formal and informal work, and household obligations. Clients waited hours at the clinic, experiencing fatigue, pain, and hunger if they waited longer than anticipated. These experiences are consistent with recent qualitative research describing long waiting times for health services in Mozambique as a major client concern [8, 10, 16, 27]. In describing how they prepared for ANC visit days, clients mentioned arranging with family and neighbors for childcare and coverage of household chores, as well as taking days off from income generating activities. If clients were unable to make these arrangements, the ANC visit might be delayed or missed entirely. Income loss from seeking health care, particularly for other conditions requiring multiple contacts such as HIV and tuberculosis, has been identified as a barrier to care access [28–31]. However, the non-financial opportunity costs of seeking health care are less commonly discussed. This investigation highlights additional costs of multiple lengthy visits to the health centers, particularly for women whose daily obligations more often include childcare and household responsibilities [31, 32]. Experiences of women seeking ANC add to the growing research on the gendered differences in healthcare utilization, where women often bear the responsibility of frequent family planning, ANC, and early childhood care visits [31]. From a health policy and health service delivery perspective, these findings emphasize the need to alleviate non-financial costs of accessing health services, especially for women who are critical to improving maternal and child health outcomes [32].

In the intervention setting, client experiences with waiting time and the intervention illustrated how an appointment system mitigates the time costs of seeking care. Not only did it reduce the perception of the amount of time spent waiting, and in turn the time spent away from meaningful activities, but it also improved predictability. With a scheduled appointment, clients no longer had to anticipate ‘burning’ or sacrificing an entire day at the clinic. They described being able to better plan their day around the clinic visit, which allowed them to resume their responsibilities before and after their appointment and rely less on the aid of others. These findings complement the quantitative findings that showed increased ANC uptake with the scheduling intervention, suggesting that scheduling better enables clients who intend to attend their ANC appointments to actually do so [19]. Where existing research has explored the effect of reducing financial costs of care-seeking on service uptake, such as through cash transfers and vouchers, this study contributes novel findings on how client experiences with time costs can influence the uptake of care [33, 34]. The potential for reduced time costs to increase care utilization suggests that health systems should consider improving client experience as a tool to impact uptake of critical services.

In addition to providing greater insight into waiting time as a barrier to care, results revealed other findings that emphasize the need to improve convenience of care. Namely, while our research identified barriers to care such as transportation costs and negative provider experiences, which is consistent with past research in Mozambique [8, 27, 35], lack of knowledge of the importance of ANC did not emerge as a theme in our study. Rather, clients emphasized that attending ANC was a mother’s duty and perceived it as critical to the healthy development of their child. This positive shift in attitudes towards facility-based ANC, which has also been observed by Biza et al. [8], suggests that addressing ease of access to ANC appointments, especially through waiting time, could allow for improved ANC uptake.

The potential impact of the scheduling system could be moderated by client behaviors regarding late ANC initiation. In the present study, clients reported that women may wait until the second or third trimester to begin care, since that is when the pregnancy may be visually confirmed. This delaying of ANC has been a persistent challenge in Mozambique, with literature dating back to 1994 and as recent as 2016 finding the same pattern [8, 9, 16]. More research and education efforts are needed to change behaviors around when to initiate ANC, as opportunities for clients to receive critical ANC services are limited by the first visit and time until delivery.

### Limitations

One limitation of this study is that the participant samples are not population representative, and potential experiences with the scheduling system may vary in other health care settings in Mozambique. In particular, our rural sample size was not large enough for in-depth analysis of rural experiences and to enable comparison between urban and rural contexts. However, while not encompassing of all possible experiences, we aimed to capture a variety of experiences to allow for a range of reflections on clients’ experiences with the scheduling intervention [24].

Additionally, no clients were interviewed who sought ANC for the first time in the eighth or ninth month of pregnancy. By nature of speaking with clients already at the clinic, it was not possible to interview clients who did not seek ANC at all. However, clients who fit these criteria are rare (only 6.7% of women report no ANC) [22], and should not compromise the validity of findings pertaining to typical care seeking behavior.

Finally, despite efforts to emphasize confidentiality and encourage open discussion, participants may have been reluctant to criticize the quality of care received in facilities or because interviews took place at the clinic and with researchers affiliated with the Ministry of Health [36].

## Conclusion

Waiting time poses a barrier to care for clients seeking ANC over multiple visits. While clients are motivated to attend ANC appointments, early morning arrivals and hours spent waiting take time away from other obligations such as childcare, household responsibilities, and income generating activities. A scheduling intervention that reduces waiting time improves the client experience by allowing more predictable planning for ANC and more time to fulfill responsibilities other than ANC. Simple interventions to schedule care may offer a low-cost way to improve client experience, which in turn contributes to achieving global health priorities of increased access to and use of high quality critical health interventions [2].

## Additional files

Additional file 1: **Table S1.** Client Characteristics at Baseline. Baseline participant demographic information at study sites. (DOCX 18 kb)

Additional file 2: Interview Guide (English). Interview guide for ANC clients. (DOCX 19 kb)

## Data Availability

The data generated during the current study are not publicly available due to participant confidentiality restrictions but are available from the corresponding author on reasonable request.

## Abbreviations

ANC: Antenatal Care
MCH: Maternal and Child Health
MOH: Ministry of Health

## References

[1] Bhutta Zulfiqar A, Das Jai K, Bahl Rajiv, Lawn Joy E, Salam Rehana A, Paul Vinod K, Sankar M Jeeva, Blencowe Hannah, Rizvi Arjumand, Chou Victoria B, Walker Neff (2014). Can available interventions end preventable deaths in mothers, newborn babies, and stillbirths, and at what cost?. The Lancet.

[2] World Health Organization. WHO recommendations on antenatal care for a positive pregnancy experience [Internet]. Geneva: World Health Organization; 2016 [cited 2019 Jun 22]. (WHO Guidelines Approved by the Guidelines Review Committee). Available from: http://www.ncbi.nlm.nih.gov/books/NBK409108/.

[3] dos Anjos Luis A, Cabral P. Geographic accessibility to primary healthcare centers in Mozambique. Int J Equity Health [Internet]. 2016;15(1):173. Available from: 10.1186/s12939-016-0455-0 10.1186/s12939-016-0455-0 PMC5070361 27756374

[4] Long Q, Madede T, Parkkali S, Chavane L, Sundby J, Hemminki E. Maternity care system in Maputo, Mozambique: Plans and practice? Cogent Med [Internet]. 2017 Jan 1 [cited 2018 Apr 29];4(1):1412138. Available from: https://www.cogentoa.com/article/10.1080/2331205X.2017.1412138

[5] Dgedge M, Mendoza A, Necochea E, Bossemeyer D, Rajabo M, Fullerton J. Assessment of the nursing skill mix in Mozambique using a task analysis methodology. Hum Resour Health [Internet]. 2014 Jan 25 [cited 2019 Jun 16];12(1):5. Available from: 10.1186/1478-4491-12-5 10.1186/1478-4491-12-5 PMC3909508 24460789

[6] United Nations Inter-agency Group for Child Mortality Estimation (UN IGME) (2018). Levels & Trends in child mortality: report 2018, estimates developed by the United Nations inter-agency Group for Child Mortality Estimation.

[7] WHO, UNICEF, UNFPA, World Bank Group and the United Nations Population Division. Trends in maternal mortality: 1990 to 2015: estimates by WHO, UNICEF, UNFPA, World Bank Group and the United Nations Population Division [Internet]. Geneva: World Health Organization; 2015 Nov p. 12. Report No.: WHO/RHR/15.23. Available from: https://www.who.int/reproductivehealth/publications/monitoring/maternal-mortality-2015/en/

[8] Biza A, Jille-Traas I, Colomar M, Belizan M, Requejo Harris J, Crahay B, et al. Challenges and opportunities for implementing evidence-based antenatal care in Mozambique: a qualitative study. BMC Pregnancy Childbirth [Internet] 2015;15:200–200. Available from: https://www.ncbi.nlm.nih.gov/pubmed/26330022. 10.1186/s12884-015-0625-x PMC4557743 26330022

[9] Chapman Rachel R. (2003). Endangering safe motherhood in Mozambique: prenatal care as pregnancy risk. Social Science & Medicine.

[10] Chavane L, Merialdi M, Betrán AP, Requejo-Harris J, Bergel E, Aleman A, et al. Implementation of evidence-based antenatal care in Mozambique: a cluster randomized controlled trial: study protocol. BMC Health Serv Res [Internet]. 2014;14:228–228. Available from: https://www.ncbi.nlm.nih.gov/pubmed/24886392 10.1186/1472-6963-14-228 PMC4057585 24886392

[11] Betrán Ana Pilar, Bergel Eduardo, Griffin Sally, Melo Armando, Nguyen My Huong, Carbonell Alicia, Mondlane Santos, Merialdi Mario, Temmerman Marleen, Gülmezoglu A Metin, Aleman Alicia, Althabe Fernando, Biza Adriano, Crahay Beatrice, Chavane Leonardo, Colomar Mercedes, Delvaux Therese, Dique Ali Ussumane, Fersurela Lucio, Geelhoed Diederike, Jille-Taas Ingeborg, Malapende Celsa Regina, Langa Célio, Osman Nafissa Bique, Requejo Jennifer, Timbe Geraldo (2018). Provision of medical supply kits to improve quality of antenatal care in Mozambique: a stepped-wedge cluster randomised trial. The Lancet Global Health.

[12] Akachi Y, Kruk ME (2016). Quality of care: measuring a neglected driver of improved health. Bull World Health Organ.

[13] Chemir F, Alemseged F, Workneh D. Satisfaction with focused antenatal care service and associated factors among pregnant women attending focused antenatal care at health centers in Jimma town, Jimma zone, south West Ethiopia; a facility based cross-sectional study triangulated with qualitative study. BMC Res Notes [Internet]. 2014;7:164–164. Available from: https://www.ncbi.nlm.nih.gov/pubmed/24646407 10.1186/1756-0500-7-164 PMC3994781 24646407

[14] Mrisho M, Obrist B, Schellenberg JA, Haws RA, Mushi AK, Mshinda H, et al. The use of antenatal and postnatal care: perspectives and experiences of women and health care providers in rural southern Tanzania. BMC Pregnancy Childbirth [Internet]. 2009;9:10–10. Available from: https://www.ncbi.nlm.nih.gov/pubmed/19261181 10.1186/1471-2393-9-10 PMC2664785 19261181

[15] Mahiti Gladys Reuben, Mkoka Dickson Ally, Kiwara Angwara Dennis, Mbekenga Columba Kokusiima, Hurtig Anna-Karin, Goicolea Isabel (2015). Women's perceptions of antenatal, delivery, and postpartum services in rural Tanzania. Global Health Action.

[16] Napúa Manuel, Pfeiffer James T., Chale Falume, Hoek Roxanne, Manuel Joao, Michel Cathy, Cowan Jessica G., Cowan James F., Gimbel Sarah, Sherr Kenneth, Gloyd Stephen, Chapman Rachel R. (2016). Option B+ in Mozambique. JAIDS Journal of Acquired Immune Deficiency Syndromes.

[17] Ameh S, Klipstein-Grobusch K, D’ambruoso L, Kahn K, Tollman SM, Gómez-Olivé FX (2017). Quality of integrated chronic disease care in rural South Africa: user and provider perspectives. Health Policy Plan [Internet].

[18] Kruk Margaret E, Pate Muhammad, Mullan Zoë (2017). Introducing The Lancet Global Health Commission on High-Quality Health Systems in the SDG Era. The Lancet Global Health.

[19] Steenland M, Dula J, de Albuquerque A, Fernandes Q, Cuco RM, Chicumbe S (2019). Evaluating the relationship between waiting time and utilization of antenatal care in Mozambique: a pre-post intervention study with a control.

[20] ICF. The DHS program STATcompiler. Funded by USAID. [internet]. 2015 [cited 2019 Jun 20]. Available from: http://www.statcompiler.com.

[21] ICF. The DHS program STATcompiler. Funded by USAID. [internet]. 2011 [cited 2019 Jun 20]. Available from: http://www.statcompiler.com.

[22] Ministério da Saúde - MISAU, Instituto Nacional de Estatística - INE, ICF. Inquérito de indicadores de imunização, malária e HIV/SIDA em Moçambique (IMASIDA) 2015 [Internet]. Maputo, Moçambique: MISAU/Moçambique, INE, and ICF; 2018 [cited 2018 Dec 16]. Available from: https://dhsprogram.com/publications/publication-AIS12-AIS-Final-Reports.cfm.

[23] Palinkas Lawrence A., Horwitz Sarah M., Green Carla A., Wisdom Jennifer P., Duan Naihua, Hoagwood Kimberly (2013). Purposeful Sampling for Qualitative Data Collection and Analysis in Mixed Method Implementation Research. Administration and Policy in Mental Health and Mental Health Services Research.

[24] Patton MQ (2015). Qualitative research & evaluation methods: integrating theory and practice.

[25] Charmaz K (2012). The power and potential of grounded theory. Med Sociol.

[26] QSR International Pty Ltd (2016). NVivo qualitative data analysis software.

[27] Munguambe K, Boene H, Vidler M, Bique C, Sawchuck D, Firoz T, et al. Barriers and facilitators to health care seeking behaviours in pregnancy in rural communities of southern Mozambique. Reprod Health [Internet]. 2016 Jun 8;13 Suppl 1(Suppl 1):31–31. Available from: https://www.ncbi.nlm.nih.gov/pubmed/27356968 10.1186/s12978-016-0141-0 PMC4943506 27356968

[28] Foster Nicola, Vassall Anna, Cleary Susan, Cunnama Lucy, Churchyard Gavin, Sinanovic Edina (2015). The economic burden of TB diagnosis and treatment in South Africa. Social Science & Medicine.

[29] Pillai N, Foster N, Hanifa Y, Ndlovu N, Fielding K, Churchyard G (2019). Patient costs incurred by people living with HIV/AIDS prior to ART initiation in primary healthcare facilities in Gauteng, South Africa. PLoS One.

[30] Geldsetzer Pascal, Mboggo Eric, Larson Elysia, Lema Irene Andrew, Magesa Lucy, Machumi Lameck, Ulenga Nzovu, Sando David, Mwanyika-Sando Mary, Spiegelman Donna, Mungure Ester, Li Nan, Siril Hellen, Mujinja Phares, Naburi Helga, Chalamilla Guerino, Kilewo Charles, Ekström Anna Mia, Foster Dawn, Fawzi Wafaie, Bärnighausen Till (2019). Community health workers to improve uptake of maternal healthcare services: A cluster-randomized pragmatic trial in Dar es Salaam, Tanzania. PLOS Medicine.

[31] Yeatman Sara, Chamberlin Stephanie, Dovel Kathryn (2018). Women's (health) work: A population-based, cross-sectional study of gender differences in time spent seeking health care in Malawi. PLOS ONE.

[32] Langer Ana, Meleis Afaf, Knaul Felicia M, Atun Rifat, Aran Meltem, Arreola-Ornelas Héctor, Bhutta Zulfiqar A, Binagwaho Agnes, Bonita Ruth, Caglia Jacquelyn M, Claeson Mariam, Davies Justine, Donnay France A, Gausman Jewel M, Glickman Caroline, Kearns Annie D, Kendall Tamil, Lozano Rafael, Seboni Naomi, Sen Gita, Sindhu Siriorn, Temin Miriam, Frenk Julio (2015). Women and Health: the key for sustainable development. The Lancet.

[33] Murray SF, Hunter BM, Bisht R, Ensor T, Bick D (2014). Effects of demand-side financing on utilisation, experiences and outcomes of maternity care in low- and middle-income countries: a systematic review. BMC Pregnancy Childbirth.

[34] Hunter BM, Harrison S, Portela A, Bick D (2017). The effects of cash transfers and vouchers on the use and quality of maternity care services: a systematic review. PLoS One.

[35] De Schacht C, Lucas C, Mboa C, Gill M, Macasse E, Dimande SA, et al. Access to HIV prevention and care for HIV-exposed and HIV-infected children: a qualitative study in rural and urban Mozambique. BMC Public Health [Internet]. 2014;14(1):1240. Available from: 10.1186/1471-2458-14-1240 10.1186/1471-2458-14-1240 PMC4265432 25467030

[36] Karnieli-Miller Orit, Strier Roni, Pessach Liat (2008). Power Relations in Qualitative Research. Qualitative Health Research.

