# Supplementary material for: Client experiences with antenatal care waiting times in southern Mozambique
Source: BMC Health Serv Res. 2019 Aug 1;19:538. doi: 10.1186/s12913-019-4369-6 (PMC6670125; doi:10.1186/s12913-019-4369-6)
Supplement: Supplementary file 1 — Table S1. Client Characteristics at Baseline. Baseline participant demographic information at study sites. (DOCX 18 kb) [file 12913_2019_4369_MOESM1_ESM.docx]

Additional File 1

Table S1. Client characteristics at baseline [1]

|  | Boane | Chissano | Machava II |
| --- | --- | --- | --- |
|  | (T) | (T) | (C) |
|  | Urban | Rural | Urban |
|  | n (%)/ | n (%)/ | n (%)/ |
|  | mean (sd) | mean (sd) | mean (sd) |
| *Client characteristics^a^* |  |  |  |
| Mean age | 23.4 (5.0) | 27.8 (7.2) | 23.5 (4.1) |
| Mean travel time to facility (mins) | 47.8 (29.9) | 45.7 (30.1) | 36.2 (23.2) |
| Mean household size | 5.2 (2.6) | 7.5 (4.8) | 4.7 (2.2) |
| Percent married | 26 (90) | 25 (89) | 22 (73) |
| Percent worked for money | 8 (28) | 22 (79) | 7 (23) |
| Percent responsible for child care^b^ | 16 (55) | 13 (46) | 11 (37) |
| N | 29 | 28 | 30 |

^a^Data from women ≥ 18 years old between 16 – 22 weeks pregnant surveyed during round one of ANC exit interviews

^b^Survey question included both the respondent’s own children and other children

References

1. Steenland M, Dula J, de Albuquerque A, Fernandes Q, Cuco RM, Chicumbe S, et al. Evaluating the relationship between waiting time and utilization of antenatal care in Mozambique: a pre-post intervention study with a control. 2019.
