# Supplementary material for: Client experiences with antenatal care waiting times in southern Mozambique
Source: BMC Health Serv Res. 2019 Aug 1;19:538. doi: 10.1186/s12913-019-4369-6 (PMC6670125; doi:10.1186/s12913-019-4369-6)
Supplement: Supplementary file 2 — Interview Guide (English). Interview guide for ANC clients. (DOCX 19 kb) [file 12913_2019_4369_MOESM2_ESM.docx]

Preamble: Thank you so much for taking the time to talk with us. As discussed in the consent form, this interview is entirely voluntary and you can stop at any time. Your privacy is very important to us, and we will do our best to protect your identity. The goal of this interview is to learn about your experience with antenatal care. There are no right or wrong answers, and you are the expert. We would like to record this interview, with your permission.

*The interview will be guided by questions of the following nature:*

| Theme | Interview Question | Probes/Notes |
| --- | --- | --- |
| Introduction | | |
| Warm up | Please tell me a little bit about yourself. | Mother’s age, residence, number of children |
| ANC beliefs | Please tell me about what brought you here today. | Probe for why women return for ANC |
| Let’s talk about going to the clinic for antenatal care | | |
| Logistics | Tell me about how you typically get to the clinic for antenatal visits. | Please tell me more about … |
|  | What are modes of transportation and costs? | Please tell me more about … |
| Decision processes | Tell me about how you decide whether you will or will not go to the clinic for an ANC visit? | Please tell me more about … |
|  | Tell me about a time you that you thought about going to the clinic but didn't. | Please tell me more about … |
|  | Tell me about how often you think women should attend ANC? | Please tell me more about … |
|  | What are reasons why you should or should not go for an ANC visit? | Probe beliefs about ANC importance |
| Advance preparation | Tell me about how you might prepare to go to the clinic. What are some challenges or things that are going well? | Probe for childcare, other responsibilities. |
| Have you attended an ANC visit here or elsewhere? If so, let’s talk about an ANC visit… | | |
| ANC Experience | Describe a typical morning when you go to the clinic for ANC. | Please tell me more about … |
|  | What time do you go? How do you get there? | Record exact times |
|  | Tell me about what you do when you get to the clinic. | Please tell me more about … |
|  | Who do you interact with? | Please tell me more about … |
|  | Tell me about how, if at all, you do or do not know when you will be seen. | Please tell me more about … |
|  | Describe what your appointments are like with the nurse. | Please tell me more about … |
|  | How do you decide whether you will or will not go back for another ANC appointment? | Please tell me more about … |
| Delivery | How do you decide where to deliver? | Please tell me more about … |
| Was one of the ANC visits at the intervention clinic? If so, let’s talk about the appointment system… | | |
| Experiences with appointment system | Do you recall getting information about when your next ANC appointment would be? | Please tell me more about … |
|  | How do you think the appointment scheduling is going? | Please tell me more about … |
|  | What was your experience at clinics before the appointment system? What was your experience after the system? | Please tell me more about … |
|  | What would you add or change about this system? | Please tell me more about … |
| Those are all our questions. Is there anything else that you would like to let us know? | | |

Thank you for your time!
